# Influence of Layer Thickness, Raster Angle, Deformation Temperature and Recovery Temperature on the Shape-Memory Effect of 3D-Printed Polylactic Acid Samples

**DOI:** 10.3390/ma10080970

**Published:** 2017-08-19

**Authors:** Wenzheng Wu, Wenli Ye, Zichao Wu, Peng Geng, Yulei Wang, Ji Zhao

**Affiliations:** School of Mechanical Science and Engineering, Jilin University, Renmin Street 5988, Changchun 130025, China; wzwu@jlu.edu.cn (W.W.); ywl16@mails.jlu.edu.cn (W.Y.); gengpeng15@mails.jlu.edu.cn (P.G.); yulei15@mails.jlu.edu.cn (Y.W.); jzhao@jlu.edu.cn (J.Z.)

**Keywords:** 3D printing, shape-memory effect, polylactic acid, orthogonal experiment

## Abstract

The success of the 3D-printing process depends upon the proper selection of process parameters. However, the majority of current related studies focus on the influence of process parameters on the mechanical properties of the parts. The influence of process parameters on the shape-memory effect has been little studied. This study used the orthogonal experimental design method to evaluate the influence of the layer thickness H, raster angle θ, deformation temperature T_d_ and recovery temperature T_r_ on the shape-recovery ratio R_r_ and maximum shape-recovery rate V_m_ of 3D-printed polylactic acid (PLA). The order and contribution of every experimental factor on the target index were determined by range analysis and ANOVA, respectively. The experimental results indicated that the recovery temperature exerted the greatest effect with a variance ratio of 416.10, whereas the layer thickness exerted the smallest effect on the shape-recovery ratio with a variance ratio of 4.902. The recovery temperature exerted the most significant effect on the maximum shape-recovery rate with the highest variance ratio of 1049.50, whereas the raster angle exerted the minimum effect with a variance ratio of 27.163. The results showed that the shape-memory effect of 3D-printed PLA parts depended strongly on recovery temperature, and depended more weakly on the deformation temperature and 3D-printing parameters.

## 1. Introduction

The shape-memory effect (SME) refers to the phenomenon in which a material can be fixed into a temporarily deformed shape and recover its original permanent shape upon the application of an external stimulus, such as heat, light, electricity or other physical or chemical factors such as magnetic or acoustic factors [1,2,3]. Polymers with this shape-memory function are called shape-memory polymers (SMPs) [4,5]. Polylactic acid (PLA) is a thermoplastic aliphatic polyester possessing superior biocompatibility and biodegradability, and it has been approved by the Food and Drug Administration (FDA) [6,7]. PLA exhibits good mechanical strength and processing ability [8,9]. Lim et al. [10] reviewed the structural, thermal, crystallization and rheological properties of PLA in relation to its converting processes. The specific process technologies such as extrusion, injection molding, casting and so on were discussed. Perego et al. [11] researched the effect of molecular weight and crystallinity on PLA’s mechanical properties. From the characterization data, poly(l-lactide) showed better mechanical properties than poly(d/l-lactide), and its behavior significantly improves with crystallization. The annealed poly(l-lactide) possessed higher values of tensional and flexural moduli of elasticity, Izod impact strength and heat resistance. PLA is also a thermosensitive SMP with a satisfactory SME. Its shape-memory process can be divided into three stages: creation of a temporary shape at a high temperature, fixation of a temporary shape at a low temperature and recovery of the permanent shape at a high temperature [12]. Sobota et al. [13] investigated the ability to control the shape memory of semicrystalline poly(l-lactide), and revealed that the SME can be controlled via modification of the material morphology. The strength of the material during permanent shape recovery increased with increasing content of crystalline phase. The rate of returning and the percent of permanent shape recovery decreased with increasing crystallinity. PLA shows potential for application in fields of vascular stent, drug delivery, surgical suture and tissue engineering scaffolds [14,15]. Senatov et al. [16] studied the mechanical properties, structural characteristics and SME of PLA/15 wt % hydroxyapatite (HA) porous scaffolds obtained by 3D printing. Dispersed particles of HA acted as nucleation centers during the ordering of PLA molecular chains, and formed an additional rigid fixed phase that reduced molecular mobility, which led to a shift of the onset of recovery stress growth from 53 to 57 °C. It was shown that PLA/15% HA porous scaffolds obtained by 3D printing with a shape recovery of 98% may be used as self-fitting implants for small bone defect replacement owing to the SME. The demonstration of a PLA spring for sutureless anastomosis utilizing the thermally-induced shape-memory effect was presented [17]. The spring was pre-expanded significantly. Upon heating to recovery temperature, it shrank to tightly hold two tubes together. The concept of a self-tightening PLA surgical staple [18], which is highly applicable in minimally invasive surgery, was demonstrated. The staple could recover its original shape under thermal excitation to complete the tightening function. The staple was designed using Solidworks and then 3D printed by MakerBot Replicator II (New York City, NY, USA), using a 1.75 mm-diameter PLA filament. A specially-designed and 3D-printed PLA spiral spring [19] was used to verify the concept of temporary endovascular embolization in interventional radiology. At high temperatures, the spring could be straightened and then stored in a catheter. Once delivered to the required position, it could be pushed out and then recover its original shape upon heating. The shape-memory effect was reported in early studies of bioabsorbable polylactic acid-based craniofacial plates [20,21]. In these studies, the plates were heated to soften the polymer, bent to a desired shape, cooled and then implanted in vitro and in vivo; these are coincidentally the exact processing conditions required to program shape memory.

Also called additive manufacturing, 3D printing is a method of manufacturing 3D solid parts by bottom-up layer-by-layer accumulation on the basis of computer-aided design and computer-aided manufacturing [22,23,24,25,26]. With the development of 3D-printing technology, 3D printing has been widely used in the aerospace, industrial design, nanosensor and biomedical fields, among others [27,28,29,30,31,32]. Fused deposition modeling (FDM) is one of the most widely used 3D-printing technologies, and uses a wire-feeding mechanism to feed the filament material into the heating nozzle. The filament is subsequently melted and extruded to manufacture parts layer-by-layer. FDM presents numerous advantages, including the variety of materials, low maintenance costs, supervision-free operation, low working temperature and so on [33,34,35]. FDM parameters include the nozzle temperature, substrate temperature, printing speed, extrusion speed, layer thickness, raster angle, build orientation, air gap, number of contours and so on [36]. The key to success in 3D printing is the proper selection of FDM parameters. Appropriate parameters can improve the printing properties of formed parts and related functions. Thus, research on the determination of the optimum parameters is an important task for production engineers [37,38,39].

4D printing is initially defined as a combination of 3D printing and time, and a combination of 3D printing and smart materials, where the shape, property, or functionality of a 3D-printed structure can change as a function of time under an external stimulus [40]. 4D printing directly builds the design into the material, simplifying the creation process from “design concept” to “physical object” [41,42]. 4D printing exhibits great application potential in the biomedical, aerospace, military and industrial fields, among others [43,44], thus drawing increasing interest from academic communities. Ge et al. [45,46] printed active composites with SMP fibers precisely printed in an elastomeric matrix, and used them as intelligent active hinges to enable origami folding patterns. By suitable thermomechanical programming, the hinges were actuated, allowing them to fold to a prescribed angle. A theoretical model was developed to guide the selection of design parameters, such as fiber dimensions, hinge lengths, programming strains and temperatures, to control the distortion of the intelligent structure. Tolley et al. [47] presented three approaches to the self-folding of structures by using low-cost, rapidly-prototyped shape-memory laminates. These structures were activated by light, heat, or electricity. They compared the fabrication of a fundamental structure (a cube) via each approach and evaluated the methods of controlling fold angles in each case. The advantages and disadvantages of each approach were also discussed. Felton et al. [48] applied 3D-printed SMP technology to produce smart structures with self-assembly and self-folding capabilities.

The majority of the current related studies focus on the influence of process parameters on the mechanical properties of the parts. By contrast, studies on the influence of process parameters on the shape-memory effect, dynamic mechanical properties, thermodynamic properties and other properties are rarely conducted. In the current study, the effects of layer thickness, raster angle, deformation temperature and recovery temperature on the shape-recovery ratio and the maximum shape-recovery rate of FDM-printed PLA were investigated by orthogonal experimental design. The extent of the effects of the experimental factors on the experimental results, and the optimal levels of the experimental factors, are discussed.

## 2. Experimental Section

### 2.1. Experimental Material

The PLA used in this study was purchased from Shenzhen Esond Technology Co., Ltd (Shenzhen, China). To determine the phase-transition temperature, energy storage modulus and other parameters of the experimental materials, the dynamic–mechanical properties of PLA were characterized using a DMA Q800 dynamic–mechanical analyzer manufactured by TA Instruments (New Castle, PA, USA).

### 2.2. Experimental Facilities

The PLA samples were fabricated by an EinStart-S 3D printer (Shining 3D, Hangzhou, China). The flexural deformation for the PLA samples with different parameters was conducted on a customized flexural experimental device. Deformation and recovery of the PLA samples at different temperatures were conducted in a constant-temperature numerical control water-bath box, and the shape-recovery experiment process was recorded with a digital camera at equal intervals.

### 2.3. Parameter Selection

Shape-memory functionalization can be realized for polymer-based materials with an appropriate morphology by the application of a specific shape-memory creation procedure (SMCP). The SME of the material is affected by many factors, including composition, applied deformation, deformation rate, deformation temperature and recovery temperature. Specifically, the temperature parameters are closely related to SME. In this study, not only two temperature parameters—namely, deformation temperature and recovery temperature—were chosen to be determined, but the layer thickness and raster angle were also determined. The effects of four parameters—deformation temperature, recovery temperature, layer thickness and raster angle—on the SME of FDM-printed PLA were evaluated.

#### 2.3.1. Deformation Temperature T_d_

In SMCP, the deformation temperature is the temperature applied during deformation of materials. The sample is heated to the deformation temperature and the temporary shape is deformed from the original shape by applying external force. Different deformation temperatures exert different effects on the phase structure of the materials, thereby influencing shape-recovery properties, such as the shape-recovery ratio and shape-recovery rate. According to the glass transition temperature of the PLA used in this experiment, two temperature levels above the glass transition temperature and two levels below the glass transition temperature were selected: 55, 60, 65 and 70 °C.

#### 2.3.2. Recovery Temperature T_r_

The recovery temperature is the temperature applied during recovery of materials. The original shape of the sample is gradually restored, from its temporary shape when the sample is heated, to the recovery temperature. Whether the material can recover its original shape, shape-recovery ratio and shape-recovery rate is directly affected by the variation in recovery temperature. The conditions and applications of SMP are also directly affected by the recovery temperature. According to the glass transition temperature and possible application of the PLA used in this experiment, four levels of recovery temperature were selected: 55, 60, 65 and 70 °C.

#### 2.3.3. Layer Thickness H

The layer thickness refers to the thickness of each layer of material deposited by the FDM nozzle. In layer-by-layer accumulation, when the layer thickness increases, the forming accuracy of the printed sample decreases, the surface roughness increases and the outer profile is prone to the step effect. Inversely, when the layer thickness is reduced, the accuracy of the printed sample is improved, the surface roughness decreases, the printing time increases and the efficiency decreases. The choice of layer thickness depends on the nozzle diameter, material properties and molding accuracy. The experiment nozzle diameter was 0.4 mm, and four levels of common layer thickness were used: 100, 150, 200 and 300 μm.

#### 2.3.4. Raster Angle θ

The raster angle refers to the angle between the path of the nozzle and the X-axis of the printing platform during FDM. The raster angles between two adjacent layers differ by 90°. The raster angle affects the forming accuracy and the mechanical performance of the printed sample. Generally, the raster angle can be selected from 0° to 90°. Thus, four levels of raster angle were selected: 0°, 15°, 30° and 45°.

### 2.4. Preparation of Samples

**Figure 1:**
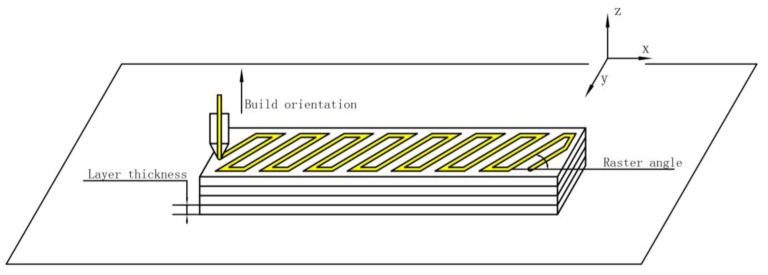
Layer thickness, raster angle and build orientation of the 3D-printed sample.

The PLA samples were fabricated by an EinStart-S 3D printer. A diagram of layer thickness, raster angle and build orientation of the 3D-printed sample is shown in Figure 1. Each experiment only changed the experimental variables, and the remaining parameters were identical. The extruder temperature was 205 °C, the printing speed was 50 mm/s and the extrusion speed was 45 mm/min. Dynamic mechanical analysis (DMA) was conducted using rectangular samples measuring 50 mm × 3 mm × 3 mm. Shape-recovery experiments were conducted using rectangular samples measuring 95 mm × 15 mm × 2.4 mm. To eliminate interference by other experimental factors, all samples were placed horizontally. At the same time, the samples were formed in the central part of the substrate to eliminate the interference caused by the difference in temperature between the position factors. To minimize the experimental error, three PLA samples were prepared in each shape-recovery experiment under the same experimental conditions.

### 2.5. Orthogonal Experimental Design

By the orthogonal experimental method, a study on the influence of deformation temperature, recovery temperature, layer thickness and raster angle on the shape-recovery ratio and maximum shape-recovery rate was conducted. To promote balanced dispersion in the orthogonal experiment, the levels of factors were randomized when experiments were arranged. Values and arrangement of four levels and four factors are shown in Table 1.

The orthogonal experimental design method is a highly efficient way of dealing with multifactor experiments and screening optimum levels by using the orthogonal design table [49]. The orthogonal experimental table is represented as L_a_(b^c^), in which L is the orthogonal array, a is the number of experiments, b is the level of factors and c is the number of columns [50]. In accordance with the requirements of the orthogonal experimental design in the experimental research, an appropriate orthogonal experimental table L_16_(4^5^) was selected for an experiment with four factors and four levels of each factor. For ANOVA analysis of an orthogonal experiment, random errors must be estimated, and a blank column was designated for the error evaluation. To improve the accuracy of the experiment and the reliability of the statistical analysis, as well as to reduce the interference of experimental error, each experiment was conducted three times. The final orthogonal experimental design is presented in Table 2.

### 2.6. Experimental Process

**Figure 2:**
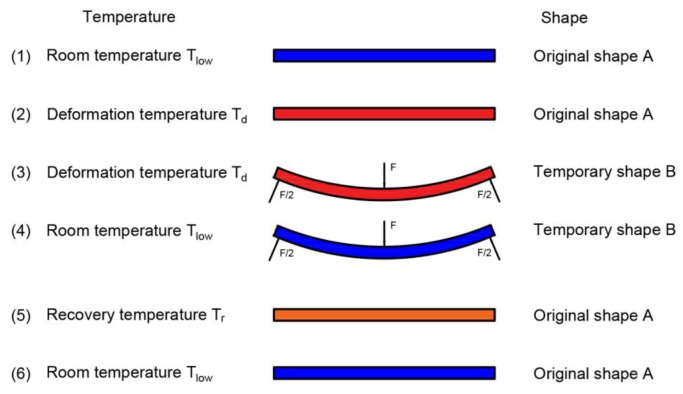
Schematic of the shape-memory effect experimental process of PLA.

In accordance with the scheme of the orthogonal experimental design, SME experiments using FDM-printed PLA samples were conducted. A schematic of the SME experimental process with PLA is presented in Figure 2.

The experimental process mainly included three parts. The first part was the deformation of the PLA sample, in which a temporary shape was created. Water in the constant-temperature numerical control water-bath box was heated to the deformation temperature. The PLA sample was placed in a water-bath box for 5 min and was then deformed to the required deflection by using a customized flexural experimental device in the water-bath box. The second part was the fixation of the deformed PLA sample, in which the temporary shape was fixed under a reduced working temperature. The temporary shape of the PLA sample remained unchanged. The PLA sample was removed from the water-bath box and then cooled down to room temperature. The third part was the recovery of the PLA sample, in which the original permanent shape of the PLA sample was restored. After the PLA sample was completely cooled and set, the water temperature in the constant-temperature numerical control water-bath box was set to the recovery temperature. The deformed PLA sample was then placed in the water-bath box, and the shape-recovery experiment was recorded every 3 s using a digital camera.

## 3. Results and Discussion

### 3.1. Dynamic Mechanical Analysis

**Figure 3:**
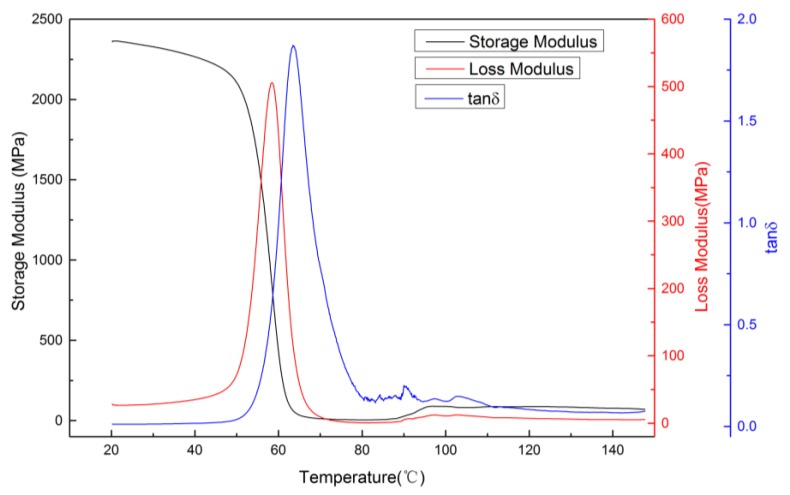
Dynamic mechanical analysis of PLA.

DMA is used to detect the relationship between temperature, frequency, stress and strain under certain experimental conditions. At the program-controlled temperature, the dynamic storage modulus, loss modulus and loss tangent of the materials under oscillatory loading are measured. DMA is widely used in the study of the viscoelastic properties of polymers. The storage modulus, loss modulus and loss tangent were evaluated and recorded under the experimental model of single-cantilever beam bending. The parameters were as follows: rate of temperature increase, 2 °C/min; scanning frequency, 1 Hz; and temperature range, 20 to 150 °C. As shown in Figure 3, the glass transition temperature (T_g_) of the PLA used in this experiment is about 63.5 °C. At temperatures ranging from about 20 to 45 °C, the PLA is in the glassy state. The storage modulus is high and relatively stable, exceeding 2000 MPa. At room temperature (25 °C), the storage modulus is about 2350 MPa. When the temperature ranges from 45 to 70 °C, the PLA is in the glass transition region, and the storage modulus of the material markedly decreases, which demonstrates the shape-memory property. At 70 °C or higher, the material is in the rubbery state, and its storage modulus is relatively low.

### 3.2. Shape-Recovery Experimental Results

**Figure 4:**
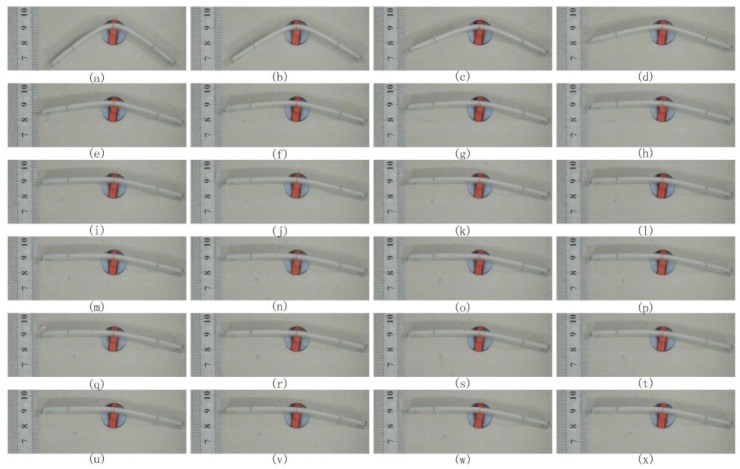
From (**a**–**x**) shows the shape-recovery process of 3D-printed PLA samples recorded by a digital camera every 3 s under the conditions of T_d_ = 55 °C, T_r_ = 65 °C, θ = 30° and H = 100 μm.

The shape-recovery process of 3D-printed PLA samples was recorded by a digital camera. The digital camera took a picture at intervals of the same time. One of the shape-recovery experimental processes of the 3D-printed PLA sample under the conditions of T_d_ = 55 °C, T_r_ = 65 °C, θ = 30° and H = 100 μm is shown in Figure 4. Figure 4a–x are photos recorded by a digital camera every 3 s. The shape-recovery ratio R_r_ and the maximum shape-recovery rate V_m_ were calculated by the following formulas.

**Figure 5:**
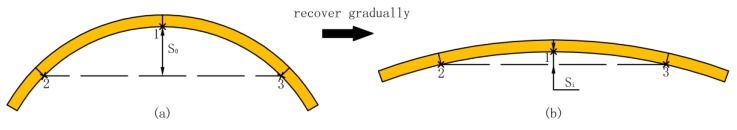
The deflection of a 3D-printed PLA sample in the shape-recovery process. (**a**) The original deflection of deformed PLA sample; (**b**) deflection during the recovery process.

The shape-recovery ratio R_r_ is the ratio of the difference between the original deformation and the recovery deformation. It is an important characteristic for the quantification of SME. The shape-recovery rate V is the recovery deformation in unit time. It is another important index to measure SME. During shape recovery, the shape-recovery rate varies, so the maximum shape-recovery rate V_m_ was used as the experimental result in this experiment. The original deflection, the recovery deflection and the final recovery deflection during the recovery process were obtained by using image-processing software. As shown in Figure 5, three points were marked on each 3D-printed PLA sample before the shape-recovery experiments. The distance from the top mark point 1 to the line that goes through mark points 2 and 3 was measured by using the image-processing software. R_r_ and V_m_ can be calculated by Formulas (1) and (2), respectively,

R_r_ = (s_o_ − s_p_)/s_o_, (1)

where R_r_ represents the shape-recovery ratio, S_o_ denotes the original deflection and S_p_ is the final recovery deflection; and

V_m_ = max[(s_i+1_ − s_i_)/t], (2)

where V_m_ represents the maximum shape-recovery rate, S_i_ indicates the recovery deflection and t represents the time interval.

The experimental results for the shape-recovery ratio and the maximum shape-recovery rate in the orthogonal experiment are listed in Table 3. R_r1_, R_r2_ and R_r3_ represent the shape-recovery ratio of the first, second and third experiment respectively. V_m1_, V_m2_ and V_m3_ represent the maximum shape-recovery rate of the first, second and third experiment respectively.

### 3.3. Range Analysis

The range analysis of the orthogonal experiment is advantageous in that it requires a small number of calculations, involves simple calculations and allows rapid analysis. The range, R, of a factor is defined as the difference between the average value of the maximum level and the average value of the minimum level. A greater range value indicates that this factor exerts a considerable influence on the experimental index and is the main factor. According to range analysis, the primary and secondary factors can be evaluated, and the optimal level of factors and the combination of these optimal levels can be determined. The range analysis results of the orthogonal experiment for the shape-recovery ratio and the maximum shape-recovery rate are listed in Table 4 and Table 5.

#### 3.3.1. Shape-Recovery Ratio

(1) Determination of combined optimal levels

The combination of the optimal levels of factors in this experiment was A_2_B_2_C_2_D_1_. To obtain the maximum shape-recovery ratio, the parameters were set as follows: deformation temperature, 55 °C; recovery temperature, 70 °C; raster angle, 45°; and layer thickness, 150 μm.

(2) Determination of primary and secondary factors

According to the magnitude of the range, R_B_ > R_A_ > R_C_ > R_D_. Among the experimental factors, the recovery temperature exerted the greatest influence, whereas the layer thickness showed the smallest influence on the shape-recovery ratio.

#### 3.3.2. Maximum Shape-Recovery Rate

(1) Determination of the combined optimal levels

The combination of the optimal levels in this experiment was A_2_B_2_C_4_D_4_. To obtain the maximum shape-recovery rate, the parameters were set as follows: deformation temperature, 55 °C; recovery temperature, 70 °C; raster angle, 15; and layer thickness, 300 μm.

(2) Determination of primary and secondary factors

According to the magnitude of the range, R_B_ > R_D_ > R_A_ > R_C_. Among the experimental factors, the recovery temperature exerted the greatest influence, whereas the raster angle showed the least influence on the maximum shape-recovery rate.

### 3.4. ANOVA Analysis

Range analysis does not quantify the data fluctuations caused by changes in experimental conditions or by experimental errors. In addition, range analysis cannot estimate the size of the experimental error; thus, adopting ANOVA analysis is necessary to compensate for the lack of range analysis. The ANOVA analysis results for the shape-recovery ratio and the maximum shape-recovery rate are presented in Table 6 and Table 7.

F represents the variance ratio of the factor. ANOVA analysis indicated that the recovery temperature exerted the most significant effect on the shape recovery ratio, and the F value was significantly higher than the other three factors. However, the influence of layer thickness on the shape-recovery ratio was minimal. The sequence of the primary and secondary influencing factors was as follows: recovery temperature, deformation temperature, raster angle and layer thickness. These results were consistent with those of the range analysis.

ANOVA analysis indicated that the recovery temperature also exerted the most significant effect on the maximum shape-recovery rate, and the F value was higher than those of the other three factors. The raster angle exerted the least influence on the maximum shape-recovery rate. The sequence of the primary and secondary experimental factors that affect the maximum shape-recovery rate was as follows: recovery temperature, layer thickness, deformation temperature and raster angle. The results were consistent with those of the range analysis.

The SME of PLA results from a combination of polymer structure and morphology, which consists of two segregated domains: the crystalline domains as the fixed phase, and the amorphous domains as the reversible phase. A full cycle of the shape-memory procedure consists of three stages of material shape: original shape, temporary shape and original shape recovery [13]. During the initial stage, physical network points such as crystals and entanglements are formed to maintain the original shape. During the second stage, when PLA is heated to the rubbery state, the mobility of the polymer chains increases. Mobile polymer chains are deformed under the force loaded outside, which causes conformational changes in the amorphous switching phase existing in the material, producing the temporary shape [16]. When the deformation reaches the desired shape, the loaded PLA is cooled down to below the glass transition temperature. Vitrification of the amorphous phase guarantees the temporary shape fixation. Polymer chains are solidified so that they cannot spontaneously revert back to the original shape. During the third stage, the material is heated again to the rubbery state, and the stretched polymer chains of the amorphous phase, limited by crystals and tightened entanglements, relax to the previous form, releasing the stored deformation energy.

The experimental results indicated that the temperature markedly influenced the shape-recovery ratio and the shape-recovery rate of the PLA samples. With decreasing deformation temperature and increasing recovery temperature, both the shape-recovery ratio and the maximum shape-recovery rate of the PLA samples increased.

**Figure 6:**
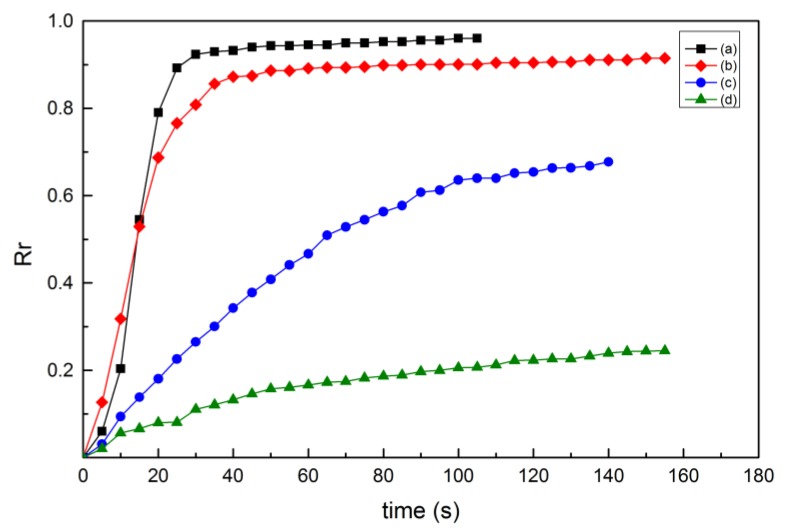
Shape-recovery ratio of 3D-printed PLA samples with time under different conditions: (**a**) T_d_ = 55 °C, T_r_ = 70 °C, θ = 0°, H = 300 μm; (**b**) T_d_ = 55 °C, T_r_ = 65 °C, θ = 30°, H = 100 μm; (**c**) T_d_ = 55 °C, T_r_ = 60 °C, θ = 45°, H = 200 μm; and (**d**) T_d_ = 55 °C, T_r_ = 55 °C, θ = 15°, H = 150 μm.

Four curves of the shape-recovery ratio with time, in the shape-recovery experiment of the PLA samples—with the same deformation temperature and different recovery temperature, raster angle and layer thickness—are shown in Figure 6. The parameters of the PLA samples are as follows: (a) T_d_ = 55 °C, T_r_ = 70 °C, θ = 0°, H = 300 μm; (b) T_d_ = 55 °C, T_r_ = 65 °C, θ = 30°, H = 100 μm; (c) T_d_ = 55 °C, T_r_ = 60 °C, θ = 45°, H = 200 μm; and (d) T_d_ = 55 °C, T_r_ = 55 °C, θ = 15° H = 150 μm. With the increase in the recovery temperature, the shape-recovery ratio and the shape-recovery rate of the PLA samples increased.

The recovery temperature had the most significant effect on the shape-recovery ratio and the maximum shape-recovery rate of the PLA samples. The PLA behaved as a physically cross-linked thermoplastic in this study. The amorphous domains of PLA are switching domains, while the crystalline domains act as permanent netpoints stabilizing the original shape [51]. From a microscale viewpoint, when the shape-recovery process is carried out at or above the glass transition temperature, the dramatic change of polymer chain mobility induced by the glass transition motivates the shape recovery of PLA [52]. The stretched polymer chains of the amorphous phase, limited by crystals and tightened entanglements, relax to the previous form, releasing the stored stress. From the thermodynamic concepts of entropy and internal energy, the fundamental of shape memory in polymer–physical networks is based on changes in the conformational entropy state [53]. The driving force for the recovery of the original shape is the entropy of elasticity of the switching chain segments, which gain entropy by moving to a random coil-like conformation. The glass transition temperature of the PLA used in this study was about 63.5 °C in accordance with DMA. When the recovery temperature was 70 or 65 °C, the PLA sample could be heated to the rubbery state with the high chain mobility so that it had sufficient energy to recover its original shape. Under the recovery temperature of 70 °C, the shape-recovery ratio could exceed 90% and the maximum could reach about 98%. However, when the recovery temperature was below the glass transition temperature—that is, 60 or 55 °C—the polymer chain mobility was simply too low to allow significant recovery. No sufficient energy was available for the sample to return to its original shape, and the shape-recovery ratio was relatively low. Under the recovery temperature of 55 °C, the shape-recovery ratio was lower than 25%; for some, it was less than 10%.

**Figure 7:**
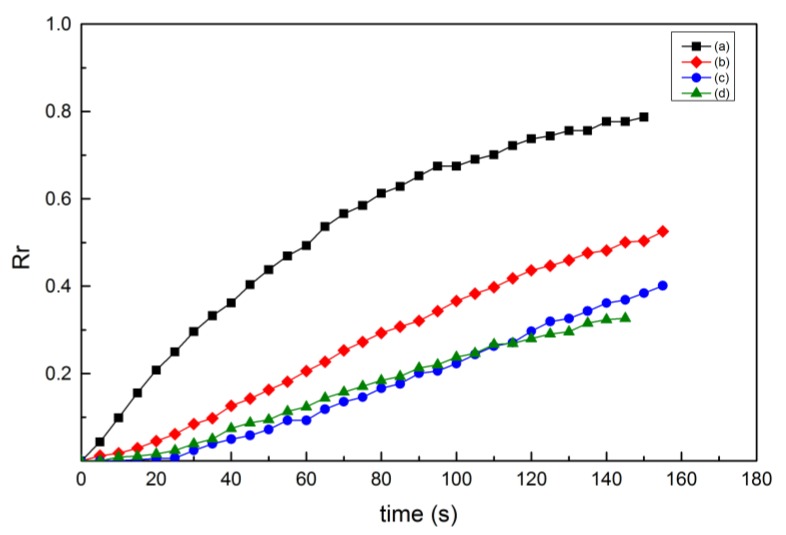
Shape-recovery ratio of 3D-printed PLA samples with time under different conditions: (**a**) T_d_ = 55 °C, T_r_ = 60 °C, θ = 45°, H = 200 μm; (**b**) T_d_ = 60 °C, T_r_ = 60 °C, θ = 0°, H = 150 μm; (**c**) T_d_ = 65 °C, T_r_ = 60 °C, θ = 30°, H = 300 μm; and (**d**) T_d_ = 70 °C, T_r_ = 60 °C, θ = 15°, H = 100 μm.

Four curves of the shape-recovery ratio with time, in the shape-recovery experiment using the PLA samples with the same recovery temperature and different deformation temperature, raster angle and layer thickness, are shown in Figure 7. The process parameters of the PLA samples were as follows: (a) T_d_ = 55 °C, T_r_ = 60 °C, θ = 45°, H = 200 μm; (b) T_d_ = 60 °C, T_r_ = 60 °C, θ = 0°, H = 150 μm; (c) T_d_ = 65 °C, T_r_ = 60 °C, θ = 30°, H = 300 μm; and (d) T_d_ = 70 °C, T_r_ = 60 °C, θ = 15°, H = 100 μm. With an increase in the deformation temperature, the shape-recovery ratio of the PLA samples decreased.

The difference in deformation temperature can cause a difference in the molecular stability of the material under the same deformation conditions. In accordance with DMA, at low temperatures, PLA obtained a high storage modulus and the material was in a low conformational-entropy state. Thus, for PLA samples to achieve the required deformation, more force was needed and more internal stress was produced in the polymer chains. PLA samples with a low deformation temperature were again heated to the rubbery state. The released stress of molecules stored in the reversible phase was greater, and the greater recovery force caused the shape-recovery ratio and the maximum shape-recovery rate to increase. Deformed at a higher temperature, the storage modulus of the PLA decreased, and the same deformation required less force. Moreover, slippage or dislocation between crystal plates of the crystalline phase could cause irreversible deformation, resulting in a reduction in the shape-recovery ratio.

Layer thickness showed the smallest influence on the shape-recovery ratio of the PLA samples; however, it exerted a greater influence on the maximum shape-recovery rate. The range analysis of the shape-recovery ratio indicated that the optimal level of layer thickness was 150 μm. For the maximum shape-recovery rate, a layer thickness of 300 μm was the optimal level. As the layer thickness increased, the number of printing layers decreased under the condition that the thickness of the sample was constant. When the layer thickness was 300 μm, the PLA sample had the fewest number of layers. The PLA sample had the minimum temperature gradient, and the heat-transfer rate from the outer layers to the inner layers was the highest. In the recovery process, under the same experimental conditions, the inner layers of the PLA warmed faster and achieved the rubbery state faster. The stress stored in the stretched polymer chains was released, and a higher shape-recovery ratio was gained. By contrast, the layer thickness was small, and the printing layer increased, resulting in a greater temperature gradient. The inner layers of the PLA sample were heated slowly. More time was required to achieve the rubbery state; thus, the shape-recovery rate was low.

The raster angle slightly influenced both the shape-recovery ratio and the maximum shape-recovery rate of the PLA sample. The raster angle exerted the smallest effect on the maximum shape-recovery rate. The raster angle of 45° was the optimal level for the shape-recovery ratio. In the printing process, the raster angle between layers was alternated with an angle of 90°. When the raster angle was 45°, the raster angle between the two adjacent layers was 45° and −45°, and each layer was deformed by bending forces, resulting in stretching of the polymer chains of each layer. More stress would be stored in the stretched polymer chains, and a higher shape-recovery ratio would be gained during shape recovery. When the raster angle was 0°, the raster angles of the two adjacent layers were 0° and 90°, respectively. Printed raster with a raster angle of 90° exhibited extremely mild deformation along the force direction, so that the expansion of the polymer chains was minimal, resulting in a decreased shape-recovery ratio during recovery.

## 4. Conclusions

3D-printed PLA samples were prepared in experiments with different layer thicknesses and raster angles. The influence of FDM parameters—namely, layer thickness and raster angle—and deformation temperature and recovery temperature in SMCP on the shape-recovery ratio and the maximum shape-recovery rate was evaluated using the orthogonal experimental design method. Range analysis and ANOVA analysis indicated that the sequence of the magnitude of effect of the four factors on the shape-recovery ratio was as follows: recovery temperature, deformation temperature, raster angle and layer thickness. The combination of optimal levels of the shape-recovery ratio was as follows: deformation temperature, 55 °C; recovery temperature, 70 °C; raster angle, 45°; and layer thickness, 150 μm. The factors affecting the maximum shape-recovery rate, in descending order, were recovery temperature, layer thickness, deformation temperature and raster angle. The combination of optimal levels of the maximum shape-recovery rate was as follows: deformation temperature, 55 °C; recovery temperature, 70 °C; raster angle, 15°; and layer thickness, 300 μm. Under different parameters, the highest shape-recovery ratio was 98%, and the maximum shape-recovery rate was 2.036 mm/s, which indicated that a large SME of the PLA showed potential for application in self-expanding vascular stents, the elimination of thrombus and in other biomedical fields. The experimental results could provide a basis for the selection of parameters in 4D printing.

## Figures and Tables

**Table 1:** Orthogonal experimental factors and levels.

	Factors	A	B	C	D
Levels		T_d_ (°C)	T_r_ (°C)	θ (°)	H (μm)
Level 1		60	60	0	150
Level 2		55	70	45	100
Level 3		65	55	30	200
Level 4		70	65	15	300

**Table 2:** Scheme of orthogonal experimental design.

	Factors	1	2	3	4	5
Test Number		T_d_ (°C)	T_r_ (°C)	θ (°)	H (μm)	Blank
1		60	60	0	150	1
2		60	70	45	100	2
3		60	55	30	200	3
4		60	65	15	300	4
5		55	60	45	200	4
6		55	70	0	300	3
7		55	55	15	150	2
8		55	65	30	100	1
9		65	60	30	300	2
10		65	70	15	200	1
11		65	55	0	100	4
12		65	65	45	150	3
13		70	60	15	100	3
14		70	70	30	150	4
15		70	55	45	300	1
16		70	65	0	200	2

**Table 3:** Experimental results of shape-recovery ratio and maximum shape-recovery rate.

	Column Number	R_r1_	R_r2_	R_r3_	V_m1_ (mm/s)	V_m2_ (mm/s)	V_m3_ (mm/s)
Test Number							
1		0.518	0.526	0.502	0.147	0.097	0.083
2		0.931	0.973	0.947	1.414	1.680	1.433
3		0.082	0.074	0.078	0.028	0.022	0.028
4		0.957	0.955	0.908	0.844	0.870	1.005
5		0.787	0.678	0.693	0.185	0.202	0.185
6		0.931	0.960	0.959	2.036	1.891	1.979
7		0.166	0.245	0.232	0.056	0.115	0.079
8		0.949	0.915	0.887	0.745	0.656	0.591
9		0.683	0.543	0.401	0.133	0.128	0.083
10		0.887	0.926	0.890	1.691	1.802	1.759
11		0.094	0.118	0.087	0.067	0.061	0.086
12		0.935	0.945	0.920	0.285	0.286	0.275
13		0.319	0.267	0.326	0.065	0.084	0.077
14		0.981	0.945	0.978	1.055	1.181	1.217
15		0.156	0.195	0.146	0.039	0.066	0.039
16		0.864	0.800	0.838	0.458	0.450	0.451

**Table 4:** Range analysis result of shape-recovery ratio.

	Column Number	1	2	3	4	5
Range		A	B	C	D	Blank
K_1j_		7.451	6.243	7.197	7.893	7.497
K_2j_		8.402	11.308	8.306	6.813	7.623
K_3j_		7.429	1.673	7.516	7.597	6.796
K_4j_		6.815	10.873	7.078	7.794	8.181
K_1j/4_		1.863	1.561	1.799	1.973	1.874
K_2j/4_		2.101	2.827	2.077	1.703	1.906
K_3j/4_		1.857	0.418	1.879	1.899	1.699
K_4j/4_		1.704	2.718	1.770	1.949	2.045
R_j_		0.397	2.409	0.307	0.270	0.346

**Table 5:** Range analysis result of maximum shape-recovery rate.

	Column Number	1	2	3	4	5
Range		A	B	C	D	Blank
K_1j_		7.651	1.469	7.806	4.876	7.715
K_2j_		8.720	19.138	6.089	6.959	6.480
K_3j_		6.656	0.686	5.867	7.261	7.056
K_4j_		5.182	6.916	8.447	9.113	6.958
K_1j/4_		1.913	0.367	1.952	1.219	1.929
K_2j/4_		2.180	4.785	1.522	1.740	1.620
K_3j/4_		1.664	0.172	1.467	1.815	1.764
K_4j/4_		1.300	1.729	2.112	2.278	1.740
R_j_		0.880	4.613	0.645	1.059	0.309

**Table 6:** ANOVA analysis results of shape-recovery ratio.

Factors	Sum of Squares, s	Degrees of Freedom, d	Variance, V	Variance Ratio, F	F_a_	Significance Level
T_d_ (°C)	0.108	3	0.036	8.780	F_0.01_(3, 35) = 4.40	**
T_r_ (°C)	5.118	3	1.706	416.100		***
θ (°)	0.077	3	0.026	6.341	F_0.05_(3, 35) = 2.88	**
H (μm)	0.060	3	0.020	4.902		**
Null error, e_1_	0.082	3				
Repeated experimental error, e_2_	0.063	32				
Total error, e	0.145	35				

*** represents the high significance level of the factor and ** represents the ordinary significance level.

**Table 7:** ANOVA analysis results of maximum shape-recovery rate.

Factors	Sum of Squares, s	Degrees of Freedom, d	Variance, V	Variance Ratio, F	F_a_	Significance Level
T_d_ (°C)	0.566	3	0.189	38.028	F_0.01_(3, 35) = 4.40	**
T_r_ (°C)	15.649	3	5.216	1049.500		***
θ (°)	0.404	3	0.135	27.163	F_0.05_(3, 35) = 2.88	**
H (μm)	0.753	3	0.251	50.503		**
Null error, e_1_	0.065	3				
Repeated experimental error, e_2_	0.109	32				
Total error, e	0.174	35				

*** represents the high significance level of the factor and ** represents the ordinary significance level.
